# Design and Construction of a Synthetic Nanobody Library: Testing Its Potential with a Single Selection Round Strategy

**DOI:** 10.3390/molecules28093708

**Published:** 2023-04-25

**Authors:** María Angélica Contreras, Yunier Serrano-Rivero, Alaín González-Pose, Julieta Salazar-Uribe, Marcela Rubio-Carrasquilla, Matheus Soares-Alves, Natalie C. Parra, Frank Camacho-Casanova, Oliberto Sánchez-Ramos, Ernesto Moreno

**Affiliations:** 1 Pharmacology Department, School of Biological Sciences, University of Concepcion, Concepcion 4070386, Chile; mcontrerasv@udec.cl (M.A.C.); malves@udec.cl (M.S.-A.); natparra@udec.cl (N.C.P.); fcamacho@udec.cl (F.C.-C.); 2 Faculty of Basic Sciences, University of Medellin, Medellin 050026, Colombia; 0905yunierserrano@gmail.com (Y.S.-R.); agonzalezp@udemedellin.edu.co (A.G.-P.); julieta.salazaru@gmail.com (J.S.-U.); marcelaru@yahoo.com (M.R.-C.)

**Keywords:** nanobody, synthetic library, phage display, CDR randomization, biopanning, tumor necrosis factor, vascular endothelial growth factor, Andes virus

## Abstract

Nanobodies (Nbs) are single domain antibody fragments derived from heavy-chain antibodies found in members of the Camelidae family. They have become a relevant class of biomolecules for many different applications because of several important advantages such as their small size, high solubility and stability, and low production costs. On the other hand, synthetic Nb libraries are emerging as an attractive alternative to animal immunization for the selection of antigen-specific Nbs. Here, we present the design and construction of a new synthetic nanobody library using the phage display technology, following a structure-based approach in which the three hypervariable loops were subjected to position-specific randomization schemes. The constructed library has a clonal diversity of 10^8^ and an amino acid variability that matches the codon distribution set by design at each randomized position. We have explored the capabilities of the new library by selecting nanobodies specific for three antigens: vascular endothelial growth factor (VEGF), tumor necrosis factor (TNF) and the glycoprotein complex (GnGc) of Andes virus. To test the potential of the library to yield a variety of antigen-specific Nbs, we introduced a biopanning strategy consisting of a single selection round using stringent conditions. Using this approach, we obtained several binders for each of the target antigens. The constructed library represents a promising nanobody source for different applications.

## 1. Introduction

Nanobodies (Nbs) are single domain antibody fragments derived from heavy-chain antibodies, lacking the light chain present in classical immunoglobulins [1]. These special antibodies are found in members of the Camelidae family, which includes camels, dromedaries, llamas and alpacas. Nbs have several important advantages as compared to antibodies and their fragments, such as their small size (~15 kDa) and high thermal stability (median melting temperature (Tm) ~67 °C [2]). These tiny proteins have found multiple applications in many different areas, from basic research—for example, as affinity capture reagents and crystallization chaperones [3])—to the clinics, with more than 40 clinical trials reported for different Nb-based products in the ClinicalTrials.gov web repository maintained by the National Institutes of Health (https://clinicaltrials.gov) and two Nbs approved for clinical use: one in the United States [4] and another one in Japan [5]. Such application versatility is due in large part to the single-domain structure of Nbs, which makes them easy to engineer and integrate into many different constructs. Notably, Nbs can achieve high affinities in spite of their smaller binding region displaying only three hypervariable loops [6].

Nbs are obtained mostly from immune libraries generated by animal immunization [6]. During the last few years, however, synthetic libraries with different designs are gaining ground as reliable Nb sources, offering important advantages in terms of cost and speed [7]. Two key features define a synthetic Nb library: framework selection and the design of the complementarity-determining regions (CDRs). A few recent works have relied on both sequence and structural data to define the CDR positions to be randomized, as well as the sets of amino acids (aa) to be introduced at those positions [8,9,10,11,12,13,14], including a recent report by our group [15].

A first, comprehensive work in designing and validating a synthetic Nb library was reported in 2016 by Moutel and coworkers [8] using as scaffold an in-house developed framework. They kept CDRs 1 and 2 with a constant length (7 aa each), randomizing each position in a way that resembles the natural diversity observed for these two CDRs. For CDR3, four different CDR3 lengths were introduced (9, 12, 15 and 18 aa) and all the positions were randomized, allowing all amino acids except cysteine. Two years later, McMahon and coworkers [9] reported the structure-based design and construction of a yeast-displayed library in which the amino acid variability in CDRs 1 (7 aa) and 2 (5 aa) recapitulates the natural diversity observed in a set of over 90 Nb crystal structures available at that time. CDR3 was constructed with different lengths (7, 11 and 15 aa), fully randomizing every position. That same year (2018), Zimmermann et al. [10] reported the design and construction of a ribosome-displayed library composed of three sub-libraries with different CDR3 lengths, using two different frameworks. Their design was based on a structure-based analysis of Nb crystal structures, finding that Nbs with a short CDR3 (6 aa) show a concave shape, those with an intermediate length (12 aa) show a protrusion, and those with a longer loop (16 aa) display a convex surface. CDR randomization focused on achieving an optimal balance between charged, polar, aromatic, and non-polar amino acids to keep a moderate hydrophobicity on the binding site surface. In a more recent (2021) report, Chen et al. [14] constructed a ribosome-displayed library using four CDR3 lengths (6, 9, 10 and 13 aa) and fully randomizing each CDR position. Several other synthetic nanobody libraries have been reported during the last few years following similar design strategies, as recently reviewed [7]. Library sizes range from 10^8^–10^10^ for phage-displayed libraries, and up to 10^12^ when using ribosome display [7].

An important issue to consider in Nb library design is the length of CDR3. It has been shown that nanobodies can recognize clefts and cryptic epitopes in proteins that are less accessible to conventional antibodies [16,17,18]. This important capability is due to the compact prolate shape of Nbs together with their usually long CDR3 loop that folds over the framework region, generating a convex paratope. In several cases, this effect may be enhanced by a protruding loop structure. Such convex–concave Nb–antigen interface provides an interaction surface as large as that of a two-domain antibody paratope, while interacting with a smaller section of the antigen [16]. As observed by Zimmermann and coworkers [10] from the analysis of a large number of nanobody crystal structures, medium length CDR3 loops (10–12 aa) adopt an extended, protruding conformation that can be inserted into a receptor cavity.

Here, we describe the design and construction of a new synthetic nanobody library with a 10 amino acid-long CDR3, in which the three hypervariable loops were subjected to position-specific randomization schemes. The design follows a structure-based approach that seeks to maintain the high stability shown by the original framework-donor nanobody and increase the number of functional variants within the combinatorial space of mutations. As scaffold, we used the framework region from the camelid nanobody cAbBCII10 [19]. This “universal” framework has been shown to be highly stable (Tm = 68 °C [20]), capable of accepting many different CDRs [21], and has been used for the construction of several Nb libraries [15,22,23,24]. The capabilities of the new library were explored by selecting nanobodies specific for three therapeutically relevant antigens: tumor necrosis factor (TNF), vascular endothelial growth factor (VEGF) and the glycoprotein complex (GnGc) of Andes virus. To test the potential of the library to yield a variety of antigen-specific Nbs, we introduced a biopanning strategy consisting of a single selection round using stringent conditions, aiming to wash out the weaker binders. By applying this strategy, we obtained several binders for each of the target antigens. For one of the obtained anti-TNF clones, we constructed a recombinant fusion protein that incorporates an albumin binding domain and confirmed the functionality of the two binding modules.

## 2. Results

### 2.1. Structure-Based Library Design

The design of this library follows a rationale similar to the approach described in a previous work by our group [15]. The amino acid sequence of the framework region was taken from the camelid nanobody cAbBCII10 [19]—a universal scaffold used for the construction of several Nb libraries [15,22,23,24]. The design of the CDRs relied on the analysis of the crystal structure of the parent cAbBCII10 nanobody (entry 3DWT in the Protein Data Bank [25]), focusing on the structural role played by individual residues in defining CDR conformation or exposing their side chains for antigen binding. The principles followed in the design of CDRs 1 and 2 are explained in detail in [15]. Briefly, the lengths of these two CDRs were kept as in the original cAbBCII10. Furthermore, the amino acids whose sidechains are packaged against framework residues in the 3D structure, as well as those found to be highly conserved in nanobody sequences, were kept as in the parent nanobody. This way we intended to preserve as much as possible the structural stability of the library mutants. CDR residues with surface-exposed side chains were subjected to tailored randomization by introducing degenerate codons in the gene sequence [26]. The allowed codons did not include cysteines and were carefully chosen to restrict the presence of hydrophobic amino acids at these solvent-exposed positions. 

**Figure 1:**
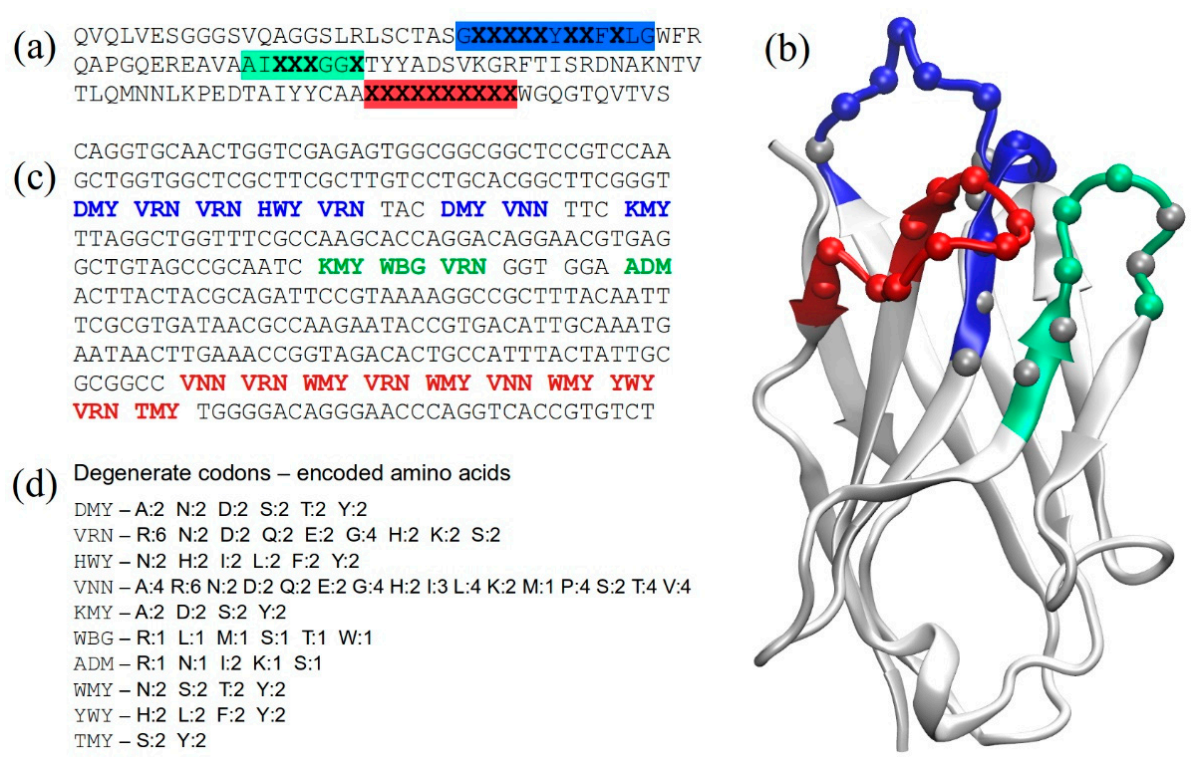
Library design. (**a**) Sequence design at the amino acid level. Positions chosen for randomization are shown as “X” in bold. The CDR sequences are highlighted in colors (blue, green and red for CDRs 1, 2 and 3, respectively), while the framework region is shown in light gray; (**b**) 3D model of a representative library nanobody based on the cAbBCII10 crystal structure (PDB: 3DWT). CDRs are colored following the same code as in panel (**a**). The colored spheres at the alpha carbons in CDRs represent the randomized positions, while gray spheres represent CDR positions that were kept fixed. (**c**) Nucleotide sequence with degenerate codons, colored by CDR; (**d**) degenerate codons used in library design and their encoded amino acids, showing also the numbers of resulting codons for each amino acid type.

For this library, we chose a 10-long CDR3, which for most of the resulting nanobody variants should create a “concave” binding site topology with an “upright”-oriented and protruding CDR3 loop. This represents an important difference as compared with our previously constructed library, carrying a 14 aa-long CDR3 that bends over the framework flank, creating a “convex” topology [15]. Codons VRN and WMY were introduced at several positions to favor the presence of polar/charged amino acids, while the relatively high probability of Gly in the VRN codon may favor a conformational diversity. The highly variable VNN codon was also used. For the C-terminal part of CDR3 (the last two residues), we took into account the amino acid frequencies observed at these positions in the crystal structures of nanobodies with short CDR3 loops, which show that Ser and Tyr are the most frequent aa at the C-terminal end (position “n”), while polar residues are frequent at position “n − 1” (our own data). For the framework region, codon usage was optimized for bacterial expression. Figure 1 shows the library design at the amino acid, nucleotide and structural levels, as well as the amino acid repertoire corresponding to each of the degenerate codons employed in the design.

A total of 22 sequence positions were randomized. The theoretical variability resulting from this tailored design (calculated by multiplying the numbers of the different amino acids coded at each randomized position) is in the order of 10^18^. This huge number, however, is in practice drastically reduced in the next two construction steps: firstly, by the actual number of genes that are synthesized and, secondly, by the number of bacteria that become transformed in the process of library construction, as explained below.

### 2.2. Library Construction 

The randomized genes were synthesized by GenScript (Piscataway, NJ, USA) and cloned as described in Methods into our ad hoc-designed pMAC phagemid vector [15] (see Figure S1). The amount of synthetic genes used for cloning (4 μg) corresponds roughly to 10^13^ individual molecules, that is, five orders of magnitude lower than the theoretical library variability. The pMAC vector employed for cloning includes a pelB leader containing a *NcoI* restriction site at its 3′ end, followed by three other unique restriction sites (*EcoRI*, *BamHI* and *NotI*, in this order). To avoid unnecessary N-terminal and/or C-terminal additions to the recombinant nanobodies, we used the outer *NcoI* and *NotI* sites for cloning. Then, the phagemid codes a short linker (SGGGG), a 6xHis tag, an amber stop codon and, finally, the M13 PIII protein. The amber codon allows the expression of recombinant nanobodies directly from recombinant library plasmids using a non-amber suppressor *E. coli* strain [27], and the obtained nanobodies can then be purified by affinity chromatography using the His tag. The library of recombinant phagemids was transformed by electroporation into SS320 *E. coli* cells as described in Methods.

The library size, which corresponds to its diversity, since with a very high probability each transformed bacterium acquired a unique nanobody gene, was assessed by colony-forming units (CFU) counting. The estimated size was 1.5 × 10^8^. Phage titration by CFU counting yielded a phage concentration of 3.6 × 10^10^ cfu/μL.

### 2.3. Assessing Library Quality and Diversity

One hundred randomly picked library clones were sequenced to evaluate the quality of the constructed library and its diversity, as compared to the theoretical design. From these clones, 76 contained a correct nanobody sequence, 15 showed a reading frame shift, 5 clones contained nanobody sequences with no CDR3, 3 clones yielded arbitrary unknown sequences and 1 clone contained an empty phagemid vector. From these results we obtain an estimate of 76% correct clones in the library, which keeps its actual size in the same order of magnitude previously determined (10^8^).

**Figure 2:**
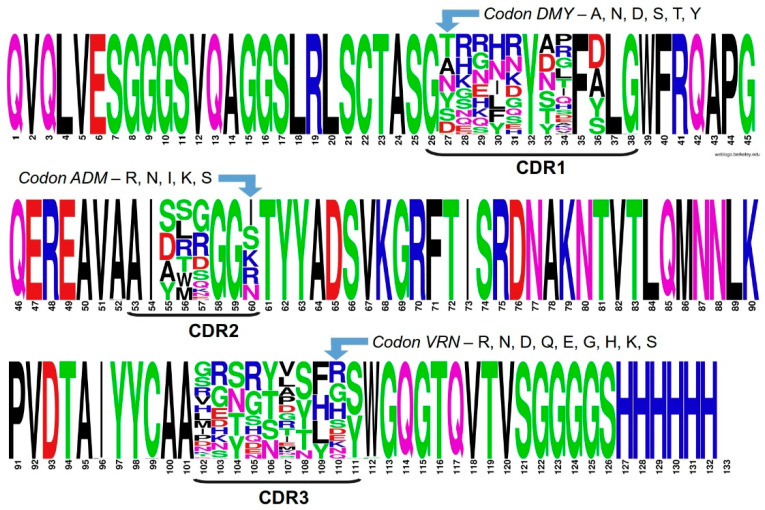
Amino acid distribution per sequence position for the ensemble of 76 correct nanobody clones, shown as a sequence logo. The framework and fixed CDR positions display their conserved amino acid as a single big letter. The amino acid variability found at each randomized position is represented as a stack of letters, each of them with a size that is proportional to its frequency in the multiple alignment. The close match between the theoretical design and the actual experimental diversity is illustrated for three CDR positions (one for each CDR).

Figure 2 shows a sequence logo obtained from the alignment of the 76 correct nanobody sequences. All the randomized positions show an amino acid variability in correspondence with the gene library design, as illustrated in the figure for three CDR positions. Furthermore, and in spite of the relatively limited number of sequenced clones, even the highly variable positions (e.g., for codons VNN and VRN) show a large diversity, matching the expected repertoire of amino acids. For example, between 14 and 16 different residues, out of 16 possible amino acids, are found at the three positions (34, 102 and 107) coded with the VNN triplet.

### 2.4. Library Screening

The capability of the library to yield specific binders was tested for three protein antigens: tumor necrosis factor (TNF), vascular endothelial growth factor (VEGF) and the glycoprotein complex (GnGc) of Andes virus. Both TNF and VEGF, as well as their receptors, are relevant therapeutic targets in cancer and autoimmune diseases, and several monoclonal antibodies targeting these molecules have been used in the clinics for several years [28,29,30]. Furthermore, several nanobodies specific for VEGF have been reported [31], and very recently (Sept/2022) a trivalent anti-TNF nanobody called ozoralizumab was approved in Japan for the treatment of rheumatoid arthritis [5]. Regarding the viral GnGc antigen, to our knowledge no nanobodies specific for this molecule have been yet reported.

#### 2.4.1. Selection of Antigen-Specific Binders in a Single Round

Here we decided to implement a screening procedure based on a single selection round using stringent conditions, aiming at a quick enrichment of the selected phages with the strongest binders in only one selection step, and also as a way of probing the capabilities of the newly designed library. Before elution, we applied four serial washes with glycine-HCl pH 2.2, a buffer commonly used for elution in phage display biopannings. No phage collection was carried out in this step since the aim of these stringent washes was to remove a large part of the phages that would bind with weaker affinity. In a subsequent, final step, the wells were incubated with a relatively high concentration of the antigen (10 µg/well, 10-fold the amount used for coating) to recover the bound recombinant phages by binding competition against the coated and soluble antigens.

**Figure 3:**
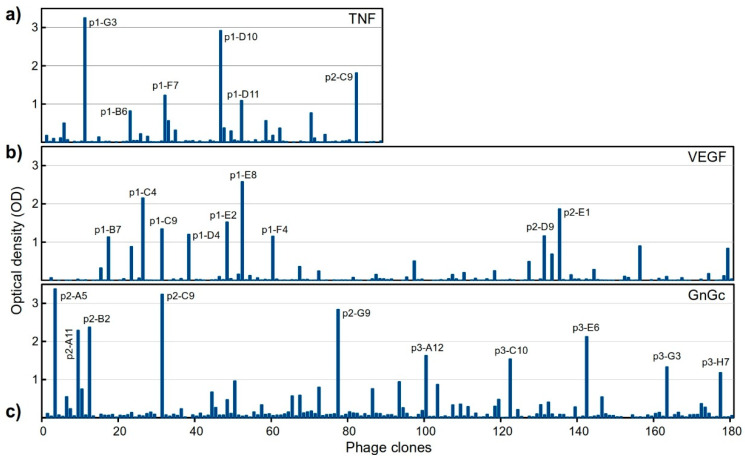
Binding of individual phage clones to their antigens, as measured by ELISA, (**a**) TNF, (**b**) VEGF, (**c**) GnGc. The optical density (OD) values for each clone corresponds to antigen binding with subtracted binding to BSA. For a few clones showing a negative value for this difference, the OD was set to 0 in the graph. The X-axis scale (clone numbers) is common for the three panels. Clones with high binding signal (OD > 1) are labeled, matching their IDs in Figure 4.

For each antigen, the whole eluted phage sample was used to infect *E. coli* TG1 bacteria, which were seeded on 2xYT/ampicillin plates. The numbers of obtained colonies were 97, 1404 and 1656 for TNF, VEGF and GnGc, respectively. We then proceeded to select individual clones to produce recombinant phages and analyze their ability to bind to their corresponding antigens. For TNF, we tested all the 97 obtained clones, whereas for both VEGF and GnGc we randomly picked 180 clones. Figure 3 shows the results from the binding experiments. Notably, we obtained a high number of positive clones in only one selection round, for the three antigens, several of them showing high OD signals.

#### 2.4.2. Sequencing of Selected Groups of Phage Clones

We decided to sequence all the clones showing OD values above 0.15, for the three antigens, resulting in 28, 24 and 44 clones for TNF, VEGF and GnGc, respectively. In practice, we obtained the sequences for 22, 22 and 34 clones, respectively, since a few of the samples could not be correctly sequenced. Nonetheless, we obtained the sequences for practically all of the best binders shown in Figure 3, with the exception of the anti-TNF clone p1-F7 and the anti-GnGc clone p2-C9.

**Figure 4:**
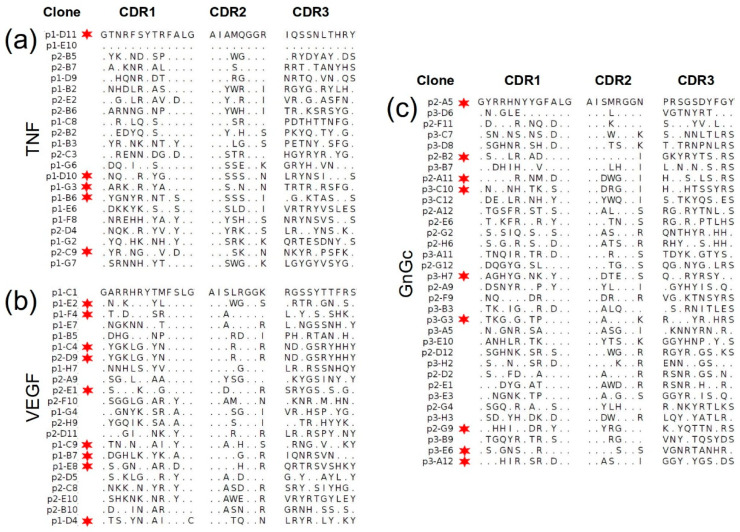
Alignments of CDR sequences obtained for groups of clones selected from the biopannings against the following: (**a**) TNF (22 clones); (**b**) VEGF (22 clones) and (**c**) GnGc (34 clones). Dots indicate the presence of the same amino acid as in the first sequence in the alignment. Red stars denote a high binding signal by ELISA (OD > 1), matching their labels in Figure 3.

As shown in Figure 4, for the three antigens we obtained sets of unique different binders (with only one identical pair of anti-TNF clones) as a consequence of performing a single selection round, without further binding clone enrichment. In the three cases, no common sequence motifs are evident from the alignment, for any of the CDRs, which suggests that these clones have different binding modes, likely recognizing different epitopes on the antigen surface.

### 2.5. Design and Expression of a Recombinant Fusion Protein with an Anti-TNF Nb

**Figure 5:**
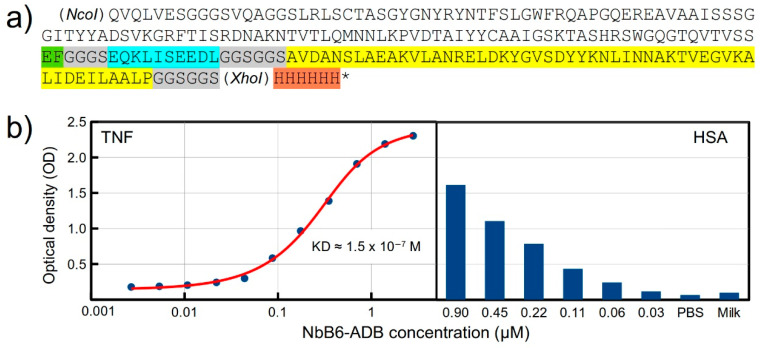
(**a**) Amino acid (aa) sequence of the NbB6-ABD fusion protein. Legend: White background —anti-TNF nanobody clone p1-B6; green—the two aa coded by an inserted *EcoRI* restriction site; gray—spacers (linkers); cyan—c-Myc tag; yellow—albumin binding domain; orange—6xHis tag. The *NcoI* and *XhoI* restriction sites were used for cloning into the pET22b vector, which adds the C-terminal histidine tag. (**b**) Binding of NbB6-ABD to TNF (**left chart**) and to HSA (**right chart**) as measured by ELISA. (**Left chart**): Negative control (not shown)—BSA coating: OD = 0.11. The red fitting curve for TNF binding was used for KD estimation. (**Right chart**): The Y-axis scale is the same as for the left chart; negative controls—PBS (instead of NbB6-ABD) and skim milk. For both antigens, we used the maximum tested NbB6-ABD concentration for the BSA/milk negative control. Experiments were performed in duplicates.

A known drawback for the therapeutic use of nanobodies is their short half-life in serum due their small size. Several strategies can be followed to prolong the Nb half-life, one of them being the genetic fusion or chemical conjugation to a molecule capable of binding to serum albumin [32]. Here, we decided to construct a fusion protein (NbB6-ABD) composed of an anti-TNF Nb and an albumin binding domain (ABD) from the *Streptococcus* sp. G protein, which shows high specificity and affinity for human serum albumin (HSA), with a dissociation constant (KD) in the nanomolar order [33,34] (Figure 5a). The anti-TNF clone p1-B6 was selected for this purpose. Although this clone is not among the strongest binders (OD = 0.9), it was chosen because of its very low background signal to BSA and skim milk (data not shown). In addition to the 46 aa constituting the ABD domain, six additional aa (AVDANS) of the protein were included at the N-terminal end since they are packed with the ABD domain in its crystal structure. A c-Myc tag was included between the nanobody and ABD, separated by short linkers. An *EcoRI* restriction site inserted right after the Nb sequence allows switching the Nb binder to target any antigen of interest. The gene coding for the fusion protein was cloned into the pET22b plasmid, which adds a C-terminal His tag, as shown in Figure 5a.

For binding assays, the fusion protein was biotinylated as described in Methods. Figure 5b shows the ELISA results for the binding to TNF and HSA. The ABD domain kept its binding capability to HSA (Figure 5b, right panel). For the binding of the nanobody domain to TNF, a titration ELISA [35] was performed in order to estimate the dissociation constant, obtaining a KD = (1.48 ± 0.35) × 10⁻⁷ M. This is quite an encouraging result, taking into account that this was an initial test for this fusion protein design, using one of the obtained anti-TNF clones, which was not among the strongest binders in the phage-based ELISAs.

## 3. Discussion

We have constructed a new synthetic nanobody library following a tailored, structure-based design. Synthetic libraries are nonspecific and therefore seek to recreate a large clonal variability to increase the probability of obtaining good binders. For this reason, synthetic libraries must be large, at least 10^8^ in size, preferably larger [6,7]. Most of the reported synthetic, phage-displayed nanobody libraries have sizes in the order of 10^9^, as recently reviewed [7]. The clonal diversity of our library is in the order of 10^8^, that is, at the lower limit of the accepted range. This level of diversity, however, proved to be enough to produce a high rate of specific clones against three different, relevant therapeutic targets. In this regard, we believe that the library’s CDR design creates a high-quality repertoire of binding paratopes that may, to a certain extent, counteract the relatively smaller size of the library.

As scaffold for the library, we chose a well-proven framework—from the cAbBCII10 Nb—that has been shown to support CDR loops of different lengths [21]. This is an important base point in the design to ensure that most of the inserted CDR sequences yield functional nanobodies. As a basis for the CDR design, firstly we carefully analyzed the structural role played by each aa in CDRs 1 and 2 in the parental cAbBCII10 Nb. A first rule applied here was to keep fixed every aa whose sidechain is buried in the structure, as well as those aa found to be highly or relatively conserved in nanobody sequences, or thought to be important in holding CDR conformation in cAbBCII10. This approach differs from the all-position randomization strategies followed in many reported libraries [7], e.g., in [8,11,13,14].

A few recent reports, however, incorporate structure-based strategies to select the positions in CDRs 1 and 2 to be randomized. For example, McMahon and coworkers [9] selected four positions in CDR1 and one in CDR2, based on their large variability in a set of analyzed Nb sequences. All these positions were fully randomized (avoiding Cys and Met). Zimmerman et al. [10] selected five residues in CDR1 and also in CDR2 for randomization, using three different mixtures of nucleotide triplets. The most used mixture coded for 18 aa (excluding Cys and Pro). CDR residues contributing to the Nb hydrophobic core were kept fixed, as in our library. By difference with these designs, here we used 10 different degenerate codons instead of triplets, tailoring the use of these codons at a position level. The amino acid repertoires resulting from these codons vary from 2 to 16 aa, with most of the sets having only 4 or 6 aa. Even so, the theoretical diversity is huge, in the order of 10^18^.

Although the cAbBCII10 Nb has been shown to accept CDR1 loops of different lengths [21], in this library we kept the full length of the parental CDR1, randomizing 8 out of its 13 positions. In contrast, only 4 positions were randomized in CDR2. Therefore, most of the variability in the library comes from CDR1 and CDR3, which in a modeled structure (Figure 1) form a shallow concave surface between them. We speculate that this shape would be likely fitting for binding to relatively small globular proteins and slightly concave surface patches on proteins in general. Furthermore, the protruding CDR3 might bind to protein cavities.

The new library was tested against three protein antigens of therapeutic relevance: TNF, VEGF and GnGc (a viral antigen). To evaluate its capabilities, we decided to apply a selection strategy consisting in applying stringent washing conditions, followed by competitive elution, aiming to retrieve mostly strong binders in a single screening step. For stringent washing (repeated four times) we used glycine-HCl pH 2.2—a commonly used elution buffer in phage display biopannings [36]. This way, many phages that otherwise would be collected for a second biopanning round were discarded. Subsequently, the wells were incubated with a relatively high concentration of antigen (100 μg/mL) to recover bound recombinant phages by binding competition against the coated antigens. Such competitive phage elution is also a common procedure used to collect phages with high affinity for their target molecule [6,37].

Stringent washes are very often used before the elution step, but in general, such stringency consists in increasing the washing time, number of washes and/or Tween 20 concentration [6,37,38,39], as well as decreasing the antigen concentration in each subsequent selection round [6,40,41]. There are few reports, however, in which a glycine-HCl solution was used as a wash buffer. Lunder et al. [42], for example, implemented several protocols that included four glycine-HCl (0.2 M, pH 2.2) washings and then eluted the phages that remained bound to the antigen by direct infection with *E. coli*, ultrasound or competition. On the other hand, although using several selection-amplification rounds enriches the library in clones specific for the target molecule, it may also have a negative effect by reducing the diversity of the finally obtained clones [37].

Here, applying stringent washes and without further enrichment rounds, we were able to obtain a significant number of clones with high, specific binding signals by ELISA, with a positivity of 13–29%. For TNF, we obtained 97 clones, all of which were tested individually. For VEGF and GnGc the number of clones was much higher—around 1400 and 1600, respectively—of which we tested only 180 in each case. Notably, all the positive clones, for the three antigens, corresponded to unique sequences (with the only exception of a pair of clones for TNF), with no evident common motifs. Since for VEGF and GnGc we tested only about 12% of the total number of clones, we would expect about a 10-fold higher number of positive clones for these two antigens, most of them most likely with unique sequences.

Finally, we tested the functionality of one of the obtained anti-TNF nanobodies, in a format of a recombinant fusion protein that incorporates an albumin binding domain—a strategy used to prolong the half-life in serum of therapeutic Nbs [32]. A similar solution was employed in the design of the anti-TNF nanobody trimer ozoralizumab (approved for clinical use in Japan), which consists of two anti-human TNF Nbs and an anti-human serum albumin Nb [5]. The estimated dissociation constant for TNF was in the order of 10^7^ M, which is an encouraging result considering that the anti-TNF clone chosen for this construction showed a moderate OD signal in the phage ELISA. This design can be also extended to multimeric Nb constructions, using Nbs targeting the same antigen in a non-competitive manner to synergically increase the affinity, as with ozoralizumab.

For future development of this library, we plan to include other CDR3 lengths to enrich its conformational variability. We are also exploring other selection strategies involving different stringent conditions and numbers of selection rounds.

## 4. Materials and Methods

### 4.1. In Silico Design and Analyses

Several bioinformatics tools were used along the design process and sequence analyses. The program VMD [43] was employed for visualization and analyses of nanobody structures. The Degenerate Codon Designer online tool (https://www.novoprolabs.com/tools/degenerate-codon-designer, NovoPro, Shanghai, China, last accessed on 20 January 2023) was used for codon analyses. The CLC Genomics Workbench v.21 (QIAGEN Aarhus, Aarhus, Denmark) was employed for sequence analyses.

### 4.2. Library Construction

The nanobody gene library was synthesized by GenScript (NJ, USA) following the theoretical design. The genes were flanked with the restriction sites *NotI* and *NcoI* for cloning in the pMAC phagemid vector [15]. After cloning (using 4 μg of both the gene library and pMAC), the recombinant plasmids were transformed by electroporation (voltage 2.5 kV, resistance 200 Ω, capacitance 25 μF) in the *E. coli* strain SS320, previously transduced with the helper phage M13KO7 (New England Biolabs, Ipswich, MA, USA).

Transformed bacteria were recovered in SOC medium for (i) determining library diversity by seeding serial dilutions in plates containing solid 2xYT medium supplemented with 100 µg/mL ampicillin, and (ii) amplifying the recombinant phage library in 2xYT medium containing 100 μg/mL ampicillin, 50 μg/mL kanamycin, 1 mM isopropyl β-d-1-thiogalactopyranoside (IPTG) by incubating 20 h at 30 °C and 185 rpm. Phage library was precipitated from the supernatant with 0.2 volumes of a solution containing PEG/NaCl (20% polyethylene glycol 8000 and 2.5 M NaCl) at 4 °C for two hours, and aliquoted in 10% glycerol until further use [44,45].

### 4.3. Library Screening

*Antigens.* Recombinant TNF [46], VEGF [47] and GnGc [48] antigens were produced and purified in-house, at the Pharmacology Department, University of Concepcion, as previously described.

Polystyrene high-binding microtiter plates (Costar) were coated with 100 µL of the antigen (TNF, VEGF or GnGc) at 10 µg/mL (24 wells per antigen for TNF and GnGc, 12 wells for VEGF), and incubated overnight at 4 °C. After two washes with phosphate buffered saline (PBS), wells were blocked with 5% skim milk (Sigma-Aldrich, Burlington, MA, USA) in PBS (300 µL/well) overnight at 4 °C. Wells were washed twice with PBS plus 0.05% Tween 20 and incubated at room temperature (RT) for two hours with 100 µL of library phages (in a quantity 500 times bigger than the library diversity) diluted in 5% skim milk. PBS plus 0.1% Tween 20 was used to perform twenty washes (250 µL/well) of five minutes each. Four additional 5 min washes were made with glycine-HCl (0.2 M, pH 2.2), and subsequently neutralized with PBS pH 7.2 for five minutes. Afterwards, recombinant phages were obtained by competitive elution with 100 µg/mL (100 µL/well) of the antigen of interest (TNF, VEGF or GnGc) for one hour at RT and 300 rpm. The E. coli strain TG1 in exponential phase of growing was transduced with the elution and incubated at 37 °C overnight in 2xYT plates supplemented with 100 µg/mL ampicillin and 2% glucose. Deep well plates were used to amplify individual clones in a final volume of 0.5 mL. Individual phage-infected colonies were picked and used to produce phagemid particles in a 96-well plate scale to test their target recognition [49].

### 4.4. Binding Assays to Detect Positive Phage Clones

Polystyrene high-binding microtiter plates (Costar) were coated with 100 µL of the antigens (TNF, VEGF or GnGc) at 5 µg/mL and incubated overnight at 4 °C. After washing with PBS, wells were blocked with 3% BSA in PBS (250 µL/well) for two hours at 37 °C. Supernatants of individual clones, previously amplified, were added to the plate (50 µL of supernatant plus 50 µL BSA 3%) for one hour at 37 °C. After three washes with PBS-0.1% Tween 20, the anti-M13 antibody conjugated to horseradish peroxidase (GE Healthcare, Chicago, IL, USA) diluted 1:5000 in BSA 1% plus PBS-0.05% Tween 20 was added for one hour at 37 °C. Plates were washed with PBS 0.1% Tween 20 and the reaction was developed with a solution of o-phenylenediamine dihydrochloride (Sigma-Aldrich) and hydrogen peroxide as substrate, and stopped with 2.5 M sulfuric acid. The absorbance was measured in a Synergy/HTX multi-mode reader (BioTek Instruments, Winooski, VT, USA) at 492 nm.

### 4.5. Sanger Sequencing

Recombinant phagemids from selected TG1 clones were purified using the GenElute Plasmid Miniprep Kit (Sigma-Aldrich) and sequenced by Macrogen (Seoul, Korea) using the standard M13R primer. Sequences were analyzed using the CLC Genomics Workbench v. 21 (QIAGEN Aarhus, Aarhus, Denmark).

### 4.6. Production of Recombinant Fusion Protein

The sequence of the ABD from the *Streptococcus* sp. G protein was taken from the PDB structure 1GJS [34]. The gene coding the chimeric protein Nb-TNFB6-ABD was synthesized and cloned into the plasmid pET22b, using the *NcoI* and *XhoI* restriction sites, by GenScript (USA). The production of NbB6-ABD was carried out in two 1L Erlenmeyers containing 500 mL each of SMM9 medium, 0.05% yeast extract (Oxoid, Basingstoke, UK), and 100 µg/mL ampicillin. After inducing the gene expression with 25 µM IPTG, the culture was stirred at 100–120 rpm and incubated at 28 °C for 18 h in a shaker-incubator (ES-20/80, BOECO, Hamburg, Germany). Next, the culture was centrifuged at 10,000× *g* for 15 min at 4 °C, the pellet was re-suspended in half-diluted SMM9, and then subjected to five freeze/thaw rounds.

Soluble NbB6-ABD was obtained in the supernatant after centrifugation at 10,000× *g* for 15 min at 4 °C. The presence of the soluble fusion protein was verified by sodium dodecyl sulphate-polyacrylamide gel electrophoresis (SDS-PAGE) and Western blot.

For SDS-PAGE, protein samples were diluted in a buffer with beta-mercaptoethanol and run in 15% polyacrylamide and 3% stacking gels. Western blot assay was performed using a 0.2 μm PVDF transfer membrane (Thermo Fisher Scientific, Waltham, MA, USA) in a semi-dry transfer system Trans-Blot^®^ Turbo™ (Bio-Rad, USA) at 0.3 A and 25 V for 30 min. After blocking with 5% skim milk in PBS, the membrane was incubated with the HRP anti-6xHis tag rabbit polyclonal antibody (ab1187, Abcam, Boston, MA, USA) diluted 1:5000 in the blocking buffer. The reaction was visualized using a DAB substrate kit (Thermo Fisher Scientific, USA).

Protein purification was performed by immobilized metal affinity chromatography (IMAC) by adding 5 mM imidazole to the equilibrium buffer (150 mM NaCl, 10 mM Na2HPO4, pH 7.7) and the initial sample diluted in the same EB. Wash and elution was done in EB by adding 25 mM and 250 mM imidazole, respectively. All fractions were monitored using the purification system BioLogic LP (BioRad, Hercules, CA, USA). Imidazole from the elution sample was removed by diafiltering against PBS (Sigma-Aldrich, Burlington, MA, USA) in 5 kDa Spin-X^®^ UF concentrators (Corning, Corning, NY, USA). Samples were analyzed by SDS-PAGE and Western blot as described above. NbB6-ABD purity was estimated using the analytical tool of the iBright 750 Imaging System (Thermo Fisher Scientific, USA), and its concentration was determined using a Pierce BCA Protein Assay Kit (Thermo Fisher Scientific, USA).

For biotinylation of NbB6-ABD, 50 µL of Na_2_CO_3_/NaHCO_3_ buffer (500 mM, pH 9.6) were mixed with 900 µL of the fusion protein (1.1 mg/mL). Next, 50 µL of biotin (H1759, Sigma-Aldrich, USA) prepared at 10 mg/mL in dimethyl sulfoxide (Merck, Rahway, NJ, USA) was slowly added at a rate of 10 µL/min and mixed. The amounts used correspond to an 80:1 biotin/NbB6-ABD molar ratio. The reaction was incubated for 6 h at room temperature (RT) under stirring. Free biotin was removed by dialysis (88244, Thermo Fisher Scientific, Waltham, MA, USA) against 4 L of 1X PBS overnight at RT.

### 4.7. Binding Assay for the Fusion Protein

Binding of biotinylated NbB6-ABD to TNF and HSA was determined by ELISA, using streptavidin-HRP (DY998, Biotechne R&D Systems, USA) for detection. Plate wells (2592, Corning, USA) were coated with 1µg of TNF or HSA in carbonate buffer pH 9.6 overnight at 4 °C, then washed three times with 0.3 mL of PBS 1X 0.1% Tween 20 (PBST) and blocked with 3% BSA or 5% milk in 1X PBS for 1 h at room temperature. Wells were then washed three times with 0.3 mL PBST and incubated for 1h at RT with 100 µL of different concentrations of the biotinylated protein. Wells were again washed three times with 0.3 mL PBST and incubated for 1 h at RT with 100 µL streptavidin-HRP (1:200, DY998, Biotechne R&D Systems, USA). They were then washed four times with 0.3 mL PBST, revealed with 100 µL of 3,3′,5,5,5′-Tetramethylbenzidine (TMB) (DY999, Biotechne R&D Systems, USA), and stopped with 50 µL of 2N H2SO4. Binding signals were read at 450 nm in a plate reader (BOECO, Germany). KD estimation was carried out followed the method and fitting function described in [35]. Linear regression analysis using this function was performed using the MyCurveFit web server (https://mycurvefit.com/, last accessed on 20 March 2023).

## Data Availability

The data presented in this study are contained in the article tables and supplementary materials.

## Supplementary Materials

The following supporting information can be downloaded at: https://www.mdpi.com/article/10.3390/molecules28093708/s1, Figure S1: Map of the designed pMAC phagemid vector.

## Sample Availability

Not applicable. The phagemids and molecules used in this work were either purchased or produced in limited amount only to perform the reported experiments.
