# Supplementary figures and images for: Design and Construction of a Synthetic Nanobody Library: Testing Its Potential with a Single Selection Round Strategy
Source: Molecules. 2023 Apr 25;28(9):3708. doi: 10.3390/molecules28093708 (PMC10180287; doi:10.3390/molecules28093708)

Figure S1. Map of the designed pMAC phagemid vector.

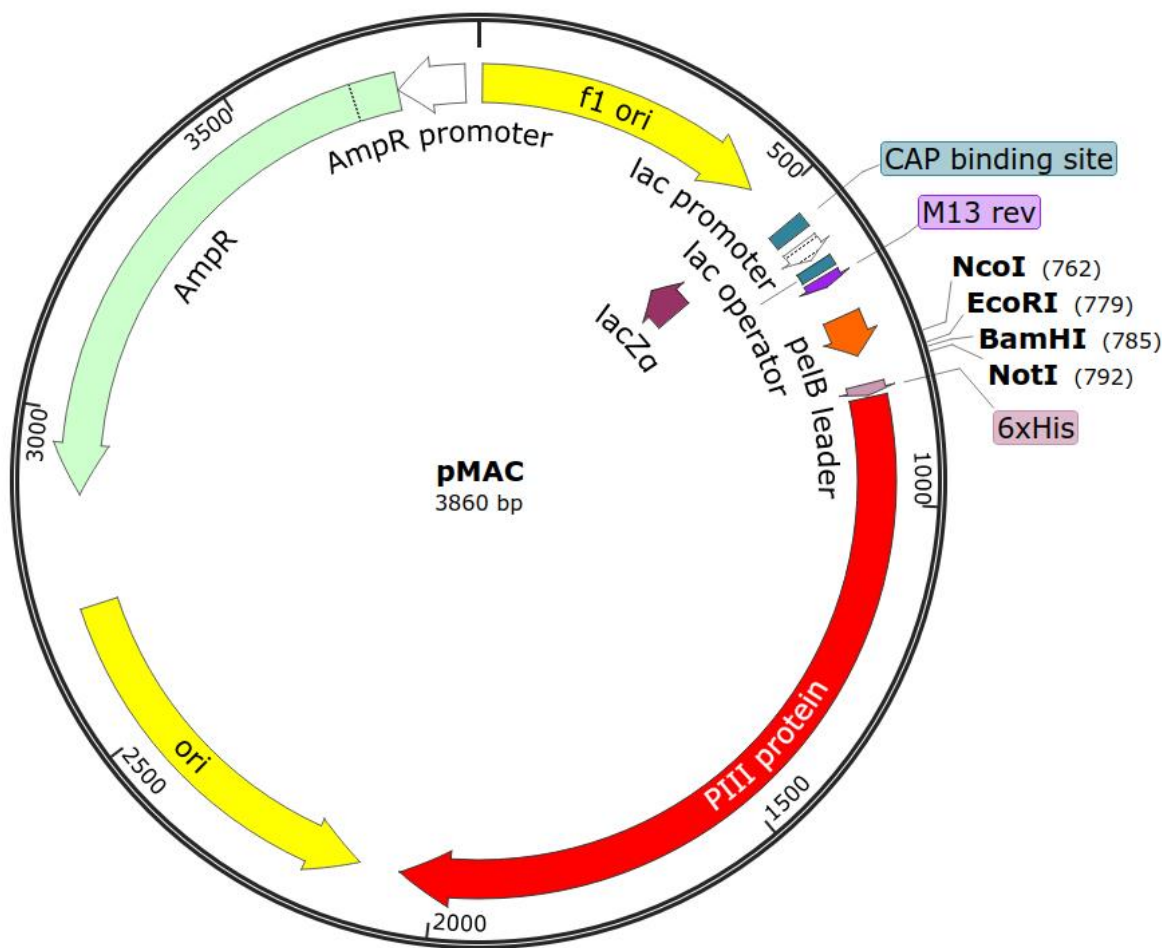

Supplement: Supplementary file 1 [file molecules-28-03708-s001.zip › molecules-2325536-SI.pdf]
